# Cross-Sectional Inverse Associations of Obesity and Fat Accumulation Indicators with Testosterone in Non-Diabetic Aging Men

**DOI:** 10.3390/ijerph15061207

**Published:** 2018-06-08

**Authors:** Iwona Rotter, Aleksandra Rył, Katarzyna Grzesiak, Aleksandra Szylińska, Wioletta Pawlukowska, Anna Lubkowska, Olimpia Sipak-Szmigiel, Krzysztof Pabisiak, Maria Laszczyńska

**Affiliations:** 1 Department of Medical Rehabilitation and Clinical Physiotherapy, Pomeranian Medical University, Żołnierska 54, 70-204 Szczecin, Poland; iwrot@wp.pl (I.R.); aleksandra.ryl@pum.edu.pl (A.R.); wpawluko@pum.edu.pl (W.P.); 2 Department of Histology and Developmental Biology, Pomeranian Medical University, Żołnierska 48, 70-204 Szczecin, Poland; kasia.grzesiak302@gmail.com (K.G.); maria@laszczynska.pl (M.L.); 3 Department of Physical Medicine and Functional Diagnostics, Pomeranian Medical University, Żołnierska 54, 70-204 Szczecin, Poland; anna.lubkowska@pum.edu.pl; 4 Department of Obstetrics and Pathology of Pregnancy, Pomeranian Medical University in Szczecin, Żołnierska 48, 71-210 Szczecin, Poland; olimpiasipak-szmigiel@wp.pl; 5 Department of Nephrology, Transplantology and Internal Medicine, Pomeranian Medical University, Powstańców Wlkp. 72, 70-111 Szczecin, Poland; bkpn@pum.edu.pl

**Keywords:** aging men, testosterone, indicators of fat accumulation, adiposity

## Abstract

*Introduction/Objective*: The aim of the study was to show which of the adipose tissue accumulation indicators correlate with testosterone disorders in non-diabetic aging men. *Material and methods*: 455 non diabetic men, recruited at primary care facilities, aged 50–75 participated in the study. The participants underwent anthropometric measurement and ELISA determination of total testosterone (TT), estradiol (E_2_), dehydroepiandrosterone sulphate (DHEA-S), sex hormone binding protein (SHBG), and the determination of fasting glucose (FPG), high-density lipids cholesterol (HDL-Ch), and triacylglycerols (TAG) in serum. The following indicators were calculated: body mass index (BMI), waist-to-hip ratio (WHR), lipid accumulation product (LAP), and visceral adiposity index (VAI). *Results*: Men with testosterone deficiency syndrome (TDS) differed in each of the assessed obesity indices from those without TDS. All of the studied parameters correlated significantly negatively with TT concentration in blood serum, with VAI being the strongest predictor of TDS. It was shown that the threshold value at which the risk of TDS increased was 28.41 kg/m^2^ for BMI, 1.58 for VAI, 104 cm for WC, and 37.01 for LAP. *Conclusions*: Indicators of fat accumulation that take into account biochemical parameters in assessing lipid metabolism are better markers of actual body fat deposition than indicators based solely on anthropometric measurements. Among them, VAI seems the most suitable biomarker of TDS in non-diabetic aging men.

## 1. Introduction

Numerous studies and meta-analyses indicate the existence of mutual relationships between low levels of testosterone (T) and obesity [1,2,3,4]. The content of adipose tissue is related to the concentration of androgen in men and affects the balance between androgens and estrogens. In 1999, Cohen presented the hypothesis of the hypogonadal-obesity cycle, which explained why the low concentration of T contributed to the accumulation of adipose tissue, and how the deposition of adipose tissue reduced the concentration of T [5]. According to that concept, T is aromatized in adipose tissue to 17-β estradiol. High expression of aromatase in adipocytes leads to a reduction in circulating T, which in turn leads to an increase in the number of adipocytes and the accumulation of adipose tissue, and so a further decrease in T levels.

The excess aromatase activity from increased adipocyte numbers in obese men results in the suppression of gonadotrophin-mediated T secretion, leading to progressive hypogonadism [3,6]. The pituitary gland, reacting to an increase in E_2_ concentration, inhibits the formation of T in the testes. A decreased T concentration leads to the accumulation of triacylglycerols (TAG) in abdominal fat tissue and to visceral obesity [7,8]. Another factor influencing that process is the effect of negative feedback from the hypothalamic-pituitary axis on T concentration, which is also regulated via aromatase [9,10].

Another factor that may be important in this process is the production of kisspeptins. These peptides, produced in the hypothalamus, modulate the secretory function of gonads and influence leptin concentration in the body. GNRH (Gonadotropin-releasing hormone) neurons in the hypothalamus possess specialized receptors for kisspeptins that produce receptors for leptin [11]. In the case of progressing obesity, adipocytes produce elevated amounts of leptin, and the hypothalamic-pituitary axis becomes resistant to leptin. Leptin also directly affects the Leydig cells in the testes, resulting in reduced production of testosterone [12].

The content and accumulation of adipose tissue, including visceral fat, can be assessed using bioimpedance, computed tomography or magnetic resonance, as well as indirectly using anthropometric measurements: BMI (body mass index), WHR (waist-hip ratio), WC (waist circumference), and indicators taking into account anthropometric measurements and biochemical parameters: LAP (lipid accumulation product) [13], VAI (visceral adiposity index) [14] and BAI (body adiposity index) [15].

The aim of the study was to show which of the indicators used in the assessment of overweight, obesity and the accumulation of adipose tissue is the best indicator of TDS (testosterone deficiency syndrome) in non-diabetic aging men. We assumed that the fat accumulation indicators that take into account biochemical parameters of lipid metabolism would be better markers of actual fat deposition in the body than those based only on anthropometric measurements. Thus, with the proven relationship between obesity and T concentration, they may be more useful in predicting hypogonadism in aging men.

## 2. Material and Methods

The study included 455 men aged between 50 and 75 who volunteered after receiving information about the course and purpose of the study from GPs from primary care facilities operating in the city of Szczecin (Poland). The study excluded patients suffering from diabetes and those in whom the glucose concentration was >126 mg/d; those undergoing oncological treatment; taking neuroleptics, antidepressants or steroids; those treated with testosterone; and people with liver and thyroid diseases, ascites and with hernia of linea alba or hernia within postoperative scars. Finally, 455 men were qualified for the study (mean age: 62.83 years ± 6.57). Written informed consent was obtained from all participating subjects. This study was performed according to the guidelines of Bioethics Committee of the Pomeranian Medical University in Szczecin (KB-0012/159/12), which abides by the Helsinki Declaration on ethical principles for medical research involving human subjects. All men participating in the study were informed about the purpose and the course of the research.

The participants underwent anthropometric measurements: body mass, body height, and abdominal circumference. Body mass index (BMI) and WHR were calculated.

Blood was collected from the ulnar vein of fasting individuals between 7:30 am to 9:00 am. Blood was collected into tubes with a clotting activator and gel separator and then centrifuged. The serum was stored at −70 °C.

In the serum, fasting plasma glucose (FPG), high-density cholesterol (HDL-Ch) and triacylglycerols (TAG) were determined using a spectrophotometric method using ready-made reagent kits (Biolabo, Aqua-Med, Łódź, Poland). Serum concentrations of hormones: total testosterone (TT), estradiol (E_2_), dehydroepiandrosterone sulphate (DHEA-S), and sex hormone binding protein (SHBG) were determined by ELISA using ready-made commercial reagent kits (DRG-MedTek, Warsaw, Poland). TDS syndrome was diagnosed according to the recommendations of the consensus of the International Society of Andrology (ISA), International Society for the Study of the Aging Male (ISSAM), European Association of Urology (EAU), the European Academy of Andrology (EAA), and the American Society of Andrology (ASA) [16]. Patients with TT below 2.5 ng/mL or between 2.5 ng/mL and 3.5 ng/mL in the presence of clinical symptoms assessed by the Morley questionnaire [17] were qualified for a TT-deficient group.

LAP was calculated using the formula: LAP = [WC (cm) − 65] × TAG (mmol/L) [13]. VAI was calculated according to the formula: VAI = WC (cm)/[39.68 + (1.88 × BMI)] × [(TAG/1.03) × (1.31/HDL-Ch)] [14].

## 3. Statistical Analysis 

Statistical analysis was performed using Statistica 13 software (StatSoft, Inc., Tulsa, OK, USA). In the characteristics of the group, the basic statistics (mean, standard deviation, minimum and maximum) were performed, and the normality of the distribution was checked using a Shapiro-Wilk test. The differences between the groups were assessed by Mann-Whitney U and Student’s *t*-tests. Correlations between the analyzed quantitative variables were calculated using a Pearson correlation coefficient. A logistic regression was used to determine the values of odds ratios (OR) and 95% confidence intervals (CI) for qualitative variables. The level of significance was assumed at *p* ≤ 0.05.

## 4. Results

Table 1 presents the parameters analyzed in all men participating in the study.

TDS was diagnosed in 182 (40%) men. Patients with TDS and those without TDS differed in each of the obesity indicators analyzed in the study (Table 2). The groups did not differ significantly in age.

Correlations between the concentration of selected hormones determined in the study and metabolic and anthropometric indicators were analyzed (Table 3). It was shown that each of the analyzed indicators correlated significantly with TT concentration in the patients’ blood serum.

WC was the only indicator showing a significant negative correlation with one of the analyzing hormone, DHEA-S.

**Figure 1:**
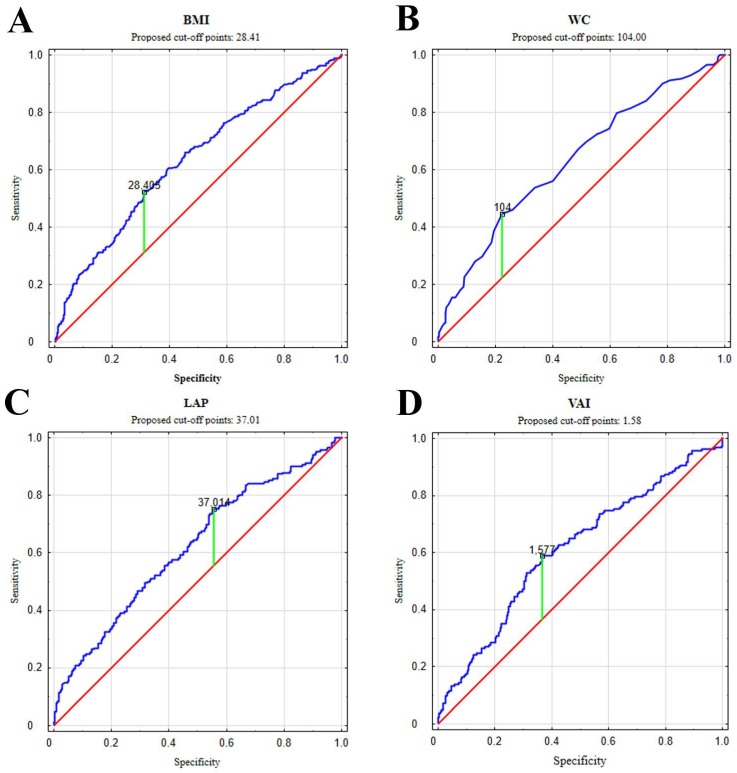
ROC curves. Cut-off points for the selected indicators of adiposity against testosterone levels: blue lines—The ROC curves for the BMI (A), WC (B), LAP (C), VAI (D), red lines—reference line.

ROC curves were analyzed by setting cut-off points for each of the analyzed indicators (Figure 1). The threshold value at which the risk of TDS increased was 28.41 kg/m^2^ for BMI, 1.58 for VAI, 104 cm for WC, and 37.01 for LAP.

## 5. Discussion

Our results indicate the existence of a relation between TDS in men and fat accumulation indicators based on simple anthropometric measurements (BMI, WC, WHR), as well as mathematical algorithms that also take into account the basic parameters of lipid metabolism (LAP, VAI).

Works available in literature primarily describe relationships between indicators based on anthropometric measurements (BMI, WC, WHR) and TT levels in men. Many studies show an inverse correlation between BMI, WHR and WC values and TT concentration, which clearly indicates that excessive fat accumulation, indirectly measured by the above-mentioned anthropometric indicators, is related to hypogonadism [18,19,20,21,22,23,24,25]. 

Osuna et al. [18] demonstrated that the concentration of TT and SHBG decreases proportionally with the increase in BMI index and the insulin resistance index. In a study similar to ours, Shamim et al. [22] showed that in healthy men aged 30–50 years a decrease in TT concentration accompanied an increase in body weight expressed in the BMI index. The results of our research indicate that this relationship also occurs in the group of men aged 50–75 years. DeFina et al. [26], in a group of 1653 men aged 50–80 years, confirmed a positive correlation between TT concentration and male fitness measured by metabolic equivalents (MET), and statistically and clinically ascertained a negative correlation between testosterone concentration and BMI index value.

Elevated BMI as well as WHR and WC values are associated with metabolic syndrome (MetS), with WC being one of the MetS criteria. Numerous studies show clear associations between MetS and age-related hypogonadism. Jaworski et al. emphasized that WC showed a stronger correlation with TDS than with BMI. The results of our research confirmed this observation. In our opinion, waist circumference is definitely a better parameter than BMI to assess obesity and—indirectly—the accumulation of adipose tissue, especially visceral fat. It should be noted that not only adipose tissue contributes to BMI level, as it also reaches higher values in the case of edema or excessive musculature.

There are few studies on the relationship between TT concentration in men and fat accumulation indicators that take into account biochemical parameters (such as VAI or LAP). There is only one publication in available literature assessing the relationship between VAI and hypogonadism [27]. In that study on Chinese men, the authors demonstrated that VAI was the best predictor of hypogonadism among the obesity indicators. This was probably due to the fact that the formula used to calculate VAI includes the greatest number of parameters that are an indirect measure of fat accumulation. Our study, conducted on a different ethnic group, confirmed that men with TDS had significantly higher VAI values. We also showed that higher levels of the LAP index, calculated with two parameters used as MetS recognition criteria, were associated with lower TT concentrations. It is difficult to put this observation in context, as in available literature we did not find any publications examining the relationship between LAP and TDS in men.

Our results showed that higher WHR and WC were associated with lower levels of DHEA-S, suggesting that abdominal obesity contributed to a reduction in this hormone. There is divergent data on this subject in literature. A recent study by Kim et al. on a group of Asian men with impaired glucose tolerance included in a diabetes prevention program did not show any significant relationship between visceral fat and DHEA-S [28]. DHEA-S also did not show a relationship with BMI in a study on Polish males between 20 and 49 years of age [29]. Similar research was conducted by Seyfart T et al. [30], who evaluated the association between sex hormones and anthropometry in a large population-based cohort, and found that testosterone levels were inversely associated with all relevant anthropometric parameters in men. On the other hand, a significant positive relationship between obesity and DHEA-S was demonstrated by Turkish researchers [31]. Their study, however, involved only 37 men. Taking into account the often recommended dehydroepiandrosterone supplementation, it is necessary to further investigate its relationship with obesity in order to draw unambiguous conclusions.

VAI and LAP are currently used as indicators of metabolic disorders, primarily to predict the risk of cardiovascular events. However, our study showed that these indicators, and in particular VAI, may also have diagnostic value in predicting disturbances in serum TT levels in healthy men. Research by Maturana et al. [32] showed that the LAP index may be associated not only with the risk of cardiovascular events (lipid profile, insulin resistance, blood pressure), but also may be related to the concentration of androgens and SHBG and cardiovascular risk factors in postmenopausal women.

It is worth noting that our research was limited by the fact that the respondents volunteered for research after obtaining information about the research program. In the absence of a random selection of the group, our results cannot be generalized, and so we plan to perform similar research on a randomly selected group of patients in the future. The applied diagnostic methods also limited our research. ELISA, which we used in our study, is much less sensitive than RIA in the determination of steroid hormones.

## 6. Conclusions

Fat collection indicators in which biochemical parameters assessing lipid metabolism are also taken into account are better markers of actual body fat deposition than those based only on anthropometric measurements. Considering the proven relationships between adiposity and hypogonadism, they may be important in suspecting and diagnosing testosterone disorders in aging men.

## Figures and Tables

**Table 1:** Characteristics of the study group (*n* = 455).

Parameters	Mean	SD	Min	Max
Age (years)	62.83	6.57	50.00	75.00
Height (m)	1.74	0.06	1.49	1.98
Body weight (kg)	83.96	13.62	51.50	136.00
BMI	27.65	4.01	18.72	43.90
VAI	1.87	1.35	0.27	9.41
WHR	0.97	0.06	0.83	1.22
WC (cm)	99.44	10.91	70.00	146.00
LAP	55.82	39.69	7.32	338.97
TAG (mmol/L)	1.54	0.78	0.43	6.65
HDL-Ch (mg/dL)	51.69	17.56	19.31	129.00
FPG (mg/dL)	90.33	18.54	57.60	126.00
TT (ng/mL)	4.05	1.71	0.09	10.15
SHBG (nmol/L)	43.44	22.08	2.58	192.00
DHEA-S (µgmL/mL)	1.14	0.90	0.01	6.57
E_2_ (pg/mL)	38.43	23.01	4.93	168.83

SD—standard deviation, Min—minimum, Max—maximum, BMI—body mass index, VAI—visceral adiposity index, LAP—lipid accumulation product, WC—waist circumference, WHR—waist-hip ratio, TAG—triglyceride, HDL-Ch—high density lipoprotein, FPG—fasting plasma glucose, TT—total testosterone, SHBG—sex hormone binding globulin, DHEA-S—dehydroepiandrosterone sulfate, E_2_—estradiol.

**Table 2:** Indicators of obesity and fat accumulation in the studied men vs testosterone concentration.

Parameters	Men without Testosterone Deficiency Syndrome (*n* = 273)				Men with Testosterone Deficiency Syndrome (*n* = 182)				*p*
	Mean	SD	Min	Max	Mean	SD	Min	Max	
**BMI**	26.68	3.58	20.01	42.28	28.98	4.04	20.02	41.32	<0.0001
**WHR**	0.99	0.06	0.84	1.22	0.97	0.06	0.83	1.14	0.354
**VAI**	1.59	1.10	0.43	7.24	2.29	1.70	0.30	9.41	<0.0001
**WC**	97.39	9.87	75.00	133.00	103.93	11.31	79.00	146.00	<0.0001
**LAP**	46.34	32.57	7.36	231.02	73.02	54.22	12.47	338.97	<0.0001

SD—standard deviation, Min—minimum, Max—maximum, *p*—statistical significance, BMI—body mass index, WHR—waist-hip ratio, VAI—visceral adiposity index, LAP—lipid accumulation product, WC—waist circumference.

**Table 3:** Correlations between the concentrations of the selected hormones and the metabolic and anthropometric indicators.

Parameters	TT		SHGB		DHEA-S		E_2_	
	P	*p*	P	*p*	P	*p*	P	*p*
**BMI**	−0.281	<0.001	0.058	0.218	−0.085	0.070	0.035	0.460
**WHR**	−0.317	<0.001	0.009	0.886	−0.122	0.049	0.122	0.051
**VAI**	−0.238	<0.001	0.020	0.663	−0.014	0.759	0.029	0.539
**WC**	−0.280	<0.001	0.003	0.951	−0.095	0.043*	0.058	0.215
**LAP**	−0.264	<0.001	0.009	0.841	−0.034	0.467	0.042	0.373

BMI—body mass index, WHR—waist-hip ratio, VAI—visceral adiposity index, LAP—lipid accumulation product, WC—waist circumference, TT—total testosterone, SHBG—sex hormone binding globulin, DHEA-S—dehydroepiandrosterone sulfate, E_2_—estradiol, P—Pearson’s linear correlation coefficient, *p*—statistical significance.
